# Layered Titanate H_2_Nd_2_Ti_3_O_10_ Intercalated With *n*-Butylamine: A New Highly Efficient Hybrid Photocatalyst for Hydrogen Production From Aqueous Solutions of Alcohols

**DOI:** 10.3389/fchem.2019.00863

**Published:** 2019-12-12

**Authors:** Ivan A. Rodionov, Elizaveta A. Maksimova, Artem Y. Pozhidaev, Sergey A. Kurnosenko, Oleg I. Silyukov, Irina A. Zvereva

**Affiliations:** Institute of Chemistry, Saint-Petersburg State University, Saint-Petersburg, Russia

**Keywords:** photocatalysis, hydrogen production, layered titanate, perovskite-type structure, intercalation, amine, hybrid compounds

## Abstract

A layered perovskite-type oxide intercalated with *n*-butylamine is reported as an efficient photocatalyst for hydrogen production from aqueous solutions of alcohols for the first time. The hybrid photocatalyst H_2_Nd_2_Ti_3_O_10_×BuNH_2_ was synthesized by solid-state ceramic method followed by protonation, intercalation of methylamine and subsequent substitution by *n*-butylamine. The product was characterized by powder XRD, TGA, STA-MS, DRS, IR, and Raman spectroscopy, CHN analysis, SEM. Intercalation of *n*-butylamine caused a dramatic increase in photocatalytic activity of H_2_Nd_2_Ti_3_O_10_ in the reaction of hydrogen evolution from aqueous solutions of methanol, ethanol, and *n*-butanol under UV radiation. While the non-intercalated Pt-loaded H_2_Nd_2_Ti_3_O_10_ showed a maximum quantum efficiency of only 2% in the 220–340 nm range, the efficiency for hybrid samples reached 23% under the same conditions and after variation of experimental parameters even 52% efficiency was achieved. This effect may be associated with the significant expansion of the interlayer space, which is considered as a separate reaction zone.

## Introduction

Excessive consumption of fuel resources and related environmental problems, which together could lead to an energy crisis, forces scientists to look for alternative energy sources, more attractive from the environmental point of view. One of the solutions to this problem is the environmentally friendly production of hydrogen fuel by splitting of water or available organic substrates using photocatalysts (Maeda, 2011). In this regard, semiconductor photocatalysts with a layered structure, especially layered perovskite-like oxides, have been intensively studied (Rodionov and Zvereva, 2016). In particular, compounds related to the Dion–Jacobson (DJ), Aurivillius (AV), and Ruddlesden–Popper (RP) phases consisting of negatively charged perovskite layers and interlayer cations with general formula A′[A_n−1_B_n_O_3n+1_] and A′_2_[A_n−1_B_n_O_3n+1_] were most actively investigated as photocatalysts (Machida et al., 2005; Compton et al., 2007; Huang et al., 2011; Chen et al., 2012; Rodionov et al., 2012, 2017a,d; Sabio et al., 2012; Zvereva and Rodionov, 2013). These materials exhibit unique photocatalytic properties derived from their layered structure, including ion-exchange and intercalation (Zvereva et al., 2011; Silyukov et al., 2015; Rodionov et al., 2017c; Shelyapina et al., 2019).

Since photocatalysts should be capable of absorbing visible light, have a large number of active catalytic sites and also support mechanisms for the efficient charge separation, different attempts have been made to modify layered oxides by cationic and anionic substitution and doping (Zou et al., 2001; Reddy et al., 2003; Kumar et al., 2011; Zhou et al., 2016; Kawashima et al., 2017a), sensitization with dyes (Youngblood et al., 2009), intercalation of metals or other inorganic particles (Huang et al., 2006, 2009) and also the creation of composites with other materials (Cui et al., 2012, 2013, 2014; Liu et al., 2018).

Hybrid organic-inorganic compounds are of great importance for modern materials science, since they allow combining the already known organic and inorganic materials and compounds with completely different properties in one composite (Gómez-Romero and Sanchez, 2006; Kickelbick, 2007). Such hybrids are interesting because the combination of components leads to improved or even new properties that were not observed in the individual components. Particularly the organic-inorganic hybrids of perovskite-like oxides can be prepared by introducing organic guests into the interlayer space while maintaining the structural features of the inorganic host. These modifications of the perovskite hosts do not require extreme conditions and lead to organic-inorganic hybrids that are stable to moderate chemical and physical impacts.

Perovskite-based hybrids are usually the result of either grafting reaction, where organic compounds are bonded to terminal interlayer oxygen atoms through strong ion-valence bonds (Tahara et al., 2007; Boykin and Smith, 2015; Shori et al., 2015), or intercalation, where organic amines form ammonium ions and interact with negatively charged perovskite layers (Tsunoda et al., 2003; Shimizu et al., 2006; Wang et al., 2007). Organic modification of layered oxide perovskites was first performed by intercalation of amines (Jacobson et al., 1987), and then expanded by covalent grafting of alcohols (Takahashi et al., 1995). Since then, the spectrum of organic compounds introduced into various protonated derivatives of the DJ, RP and AV phases has been significantly expanded (including amino alcohols, carboxylic acids, amino acids, etc.) (Hong and Kim, 1996; Han et al., 2001; Tsunoda et al., 2003; Tong et al., 2005; Takeda et al., 2006, 2008; Tahara, 2007; Wang et al., 2012, 2018; Boykin and Smith, 2015; Shori et al., 2015; Sato et al., 2017; Silyukov et al., 2018).

Despite the rather large number of currently synthesized hybrid compounds based on layered oxides, little attention has been paid to the study of the photocatalytic activity of such compounds. Perhaps this is due to concerns about the photodegradation of embedded organics during the photocatalytic process. For example, Machida et al. studied the photocatalytic activity of the layered tantalate HCa_2_Ta_3_O_10_ intercalated with C_6_H_13_NH_2_ in the reaction of water splitting under UV-light and noted a significantly higher activity of the hybrid in hydrogen evolution compared to MCa_2_Ta_3_O_10_ (M = Cs, Na, and H) (Machida et al., 2005). At the same time, the studied hybrid turned out to be unstable to oxidation during the photocatalytic process, which was confirmed by the collapse of the interlayer gallery and insignificant oxygen evolution, followed by a dramatic decrease in activity after the first 8 h of irradiation. More optimistic results were obtained by Wang et al. (2014) who used derivatives of the double-layered perovskite H_2_CaTa_2_O_7_ with n-alcohols as photocatalyst for the decomposition of rhodamine B and methyl orange under UV-visible light irradiation. Grafting of long-chain *n*-alcohols (*n* > 3) into the perovskite layers of H_2_CaTa_2_O_7_ improved the photocatalytic activity drastically. One can also note interesting results obtained for layered oxides intercalated by polyaniline. Such hybrids have shown themselves to be effective photocatalysts in the visible spectral region, having been tested on the model decomposition reaction of methylene blue dye (Guo et al., 2010; Zhu et al., 2013; Liu et al., 2014). Although aniline-intercalated compounds have also been obtained for layered perovskite-like oxides (Uma and Gopalakrishnan, 1994, 1995; Tong et al., 2005), the photocatalytic activity of such hybrids has not yet been studied.

A_2_Ln_2_Ti_3_O_10_ (A = Li, Na, K, Rb; Ln = La, Nd, Gd, Sm, Dy, Eu) are layered RP perovskite-type compounds with alkali cations in the interlayer space. These compounds are known to exhibit ion exchange and intercalation properties (Gopalakrishnan and Bhat, 1987; Richard et al., 1994; Rodionov et al., 2017d) and are one of the most extensively studied photocatalytic materials (Takata et al., 1997a,b; Rodionov et al., 2012; Kawashima et al., 2017b). Ni-loaded Rb_2_La_2_Ti_3_O_10_ was one of the first highly active photocatalysts for water splitting with a quantum efficiency estimated around 5% (Takata et al., 1997a). H_2_Ln_2_Ti_3_O_10_ are protonated forms of A_2_Ln_2_Ti_3_O_10_ compounds that can be obtained by acid leaching of interlayer alkali cations (Gopalakrishnan and Bhat, 1987; Richard et al., 1994). They can be used to develop new materials by intercalating organic molecules into the interlayer space (Tong et al., 2005; Tahara et al., 2007; Akbarian-Tefaghi and Wiley, 2018) as well as by exfoliation and self-assembly processes (Huang et al., 2006; Wu et al., 2006; Ida et al., 2008). However, to our best knowledge, amine-intercalated titanites of such structure have never been investigated as photocatalysts for hydrogen production. Particularly, it is unclear, if the intercalated organic molecules always undergo photodegradation, or, at some conditions, they might be stable and promote the photocatalytic process. Therefore, our work focuses on the photocatalytic properties of the *n*-butylamine intercalated titanate H_2_Nd_2_Ti_3_O_10_ as an example of a hybrid organic-inorganic layered material obtained by intercalation reaction. Its photocatalytic activity was investigated in the reaction of hydrogen evolution from aqueous alcohol solutions (methanol, ethanol and *n*-butanol) under UV radiation and compared with the initial H_2_Nd_2_Ti_3_O_10_ oxide.

## Materials and Methods

### Synthesis

#### K_2_Nd_2_Ti_3_O_10_

The initial layered oxide K_2_Nd_2_Ti_3_O_10_ was synthesized by conventional solid-state reaction (1) using K_2_CO_3_, Nd_2_O_3_ and TiO_2_ as reagents (Vecton, 99.9%).

(1) $$\begin{array}{l} \text{N}{\text{d}}_{2}{\text{O}}_{3}+3\text{Ti}{\text{O}}_{2}+{\text{K}}_{2}\text{C}{\text{O}}_{3}\to {\text{K}}_{2}\text{N}{\text{d}}_{2}\text{T}{\text{i}}_{3}{\text{O}}_{10}+\text{C}{\text{O}}_{2}↑ \end{array}$$

The stoichiometric amounts of oxides needed to yield 20 g of the final product (7.1497 g TiO_2_, 10.0401 g Nd_2_O_3_) with 50% excess of potassium carbonate (5.7736 g), were weighted with an accuracy of 10^−4^ g (Ohaus Pioneer PA214C balance), mixed together and ground on a Fritsch Pulverisette 7 planetary micro mill with silicon nitride accessories at a speed of 600 rpm using a program of 10 repetitions of 10 min each with 5 min interval. Hexane was also added to the bowl to prevent any hydration of the reactants and provide more uniform grinding. The obtained mixture was dried at 50°C for 1 h, then at 150°C for 30 min to remove the hexane, and finally pressed into tablets of ca. 1.5 g at 50 bar using a PI 88.00 Omec hydraulic press. The tablets were placed into corundum crucibles and heated at 700°C for 2 h in a Nabertherm L-011K2RN furnace in air atmosphere. After cooling down, the tablets were ground in an agate mortar, re-pelletized and heated at 1,000°C for 10 h as the final synthesis step. The obtained tablets were again ground in an agate mortar to prepare for XRD analysis and for the next synthesis step.

#### H_2_Nd_2_Ti_3_O_10_

Prior to protonation, the K_2_Nd_2_Ti_3_O_10_ oxide was transformed into its hydrated form K_2_Nd_2_Ti_3_O_10_×H_2_O by exposition to humid air (RH = 75%) for 24 h (Utkina et al., 2018). After the XRD analysis ensured complete hydration, the sample (15 g) was dispersed in 3 L of 0.1 M hydrochloric acid and stirred for 7 days at room temperature. Then the product was separated from the solution by centrifugation (Elmi CM-6MT centrifuge), dried in a desiccator over CaO and analyzed by XRD and TGA.

#### H_2_Nd_2_Ti_3_O_10_×MeNH_2_

To intercalate methylamine into the interlayer space of H_2_Nd_2_Ti_3_O_10_, the sample (12 g) was dispersed in 120 ml of 38% aqueous MeNH_2_ solution, sealed in a flask and stirred for 10 days at 60°C. Afterwards, the solid was separated by centrifugation, dried in air atmosphere for 2 days and analyzed by XRD, TGA, and CHN.

#### H_2_Nd_2_Ti_3_O_10_×BuNH_2_

*n*-Butylamine was introduced into the interlayer space by substitution of methylamine, because the attempts of direct intercalation into H_2_Nd_2_Ti_3_O_10_ led only to non-singe phase products. The methylamine-intercalated sample was dispersed in 80 ml of BuNH_2_ with the addition of 10 ml of water, sealed in a flask and stirred for 4 days at room temperature. The final product was separated by centrifugation, dried in air atmosphere for 2 days, characterized by the further described methods and subsequently used for photocatalytic experiments.

### Characterization

#### XRD Analysis

Powder XRD analysis was performed on every step of synthesis using a Rigaku Miniflex II diffractometer (CuKα radiation, 2θ range 3–60°, scan speed 10°/min). The obtained XRD patterns were indexed and the unit cell parameters were determined with an accuracy of 0.05% using Bruker Topas software.

#### TGA

Thermogravimetric analysis was carried out on the Netzsch TG 209 F1 Libra microbalance. In the case of H_2_Nd_2_Ti_3_O_10_, the measurement was performed in an argon atmosphere with a heating rate of 10 K/min from room temperature to 900°C. Typically, two steps of mass loss are observed on the TG curve of the protonated layered oxide H_2_Nd_2_Ti_3_O_10_ (Rodionov et al., 2017b). The first low-temperature step (RT−250°C) corresponds to the liberation of intercalated/adsorbed water (2) and the second step (300–400°C) refers to the decomposition of the protonated layered oxide with the liberation of water (3):

(2) $$\begin{array}{l} {\text{H}}_{\text{x}}{\text{K}}_{2-\text{x}}\text{N}{\text{d}}_{2}\text{T}{\text{i}}_{3}{\text{O}}_{10}\cdot \text{y}{\text{H}}_{2}\text{O}={\text{H}}_{\text{x}}{\text{K}}_{2-\text{x}}\text{N}{\text{d}}_{2}\text{T}{\text{i}}_{3}{\text{O}}_{10}+\text{y}{\text{H}}_{2}\text{O} \end{array}$$

(3) $$\begin{array}{l} {\text{H}}_{\text{x}}{\text{K}}_{2-\text{x}}\text{N}{\text{d}}_{2}\text{T}{\text{i}}_{3}{\text{O}}_{10}={\text{K}}_{2-\text{x}}\text{N}{\text{d}}_{2}\text{T}{\text{i}}_{3}{\text{O}}_{10-\text{x}/2}+\text{x}/2{\text{H}}_{2}\text{O} \end{array}$$

The protonation degree (x/2) was calculated from the mass loss at the second step using the formula

(4) $$\begin{array}{l} x=\frac{M_{K_{2}Nd_{2}Ti_{3}O_{10}}\left(m_{1}-m_{2}\right)}{m_{1}\left(M_{K}+\frac{1}{2}M_{O}\right)-m_{2}\left(M_{K}-M_{H}\right)} \end{array}$$

which can be easily derived from Equation (3). Here *m*_1_ stands for the sample mass before reaction (3) starts, *m*_2_ stands for the final mass after decomposition. *M*_*i*_ represents the molar mass of corresponding species *i* (K_2_Nd_2_Ti_3_O_10_, K, O, and H).

The TGA of amine-intercalated samples was performed in a synthetic dry air atmosphere (flow rate 100 ml/min) in order to ensure complete oxidation of organics as well as titanium, which might become partially reduced by organics at high temperature if the experiment is conducted in an inert atmosphere. The temperature program consisted of two steps: heating at a rate of 10 K/min from room temperature to 950°C followed by an isothermal step for 20 min.

#### STA-MS

Simultaneous thermal analysis coupled with the mass spectrometric detection of evolved gases (STA-MS) was carried out on a Netzsch STA 409 CD-QMS 403/5 Skimmer system using air-containing (oxidative) atmosphere (50 ml/min) at a heating rate of 20°/min.

#### CHN Analysis

The amounts of carbon, hydrogen and nitrogen in the hybrids were determined by the elemental CHN-analysis on a Euro EA3028-HT analyzer.

#### Raman Spectroscopy

Raman spectra were obtained on a Bruker Senterra spectrometer (spectral range 100–4,000 cm^−1^, laser 488 nm, 20 mW, spectrum accumulation time 10 s).

#### IR Spectroscopy

Fourier-transformed infrared (IR) absorption spectra were recorded on a Shimadzu IRAffinity-1 spectrometer (spectral range 400–4,000 cm^−1^, step 1 cm^−1^) using KBr tabletting technique.

#### DRS

Diffuse reflectance spectroscopy (DRS) was performed using a Shimadzu UV-2550 spectrophotometer with ISR-2200 integrating sphere attachment. The Kubelka-Munk function (F) was calculated by the formula

(5) $$\begin{array}{l} F=\frac{{\left(1-R\right)}^{2}}{2R} \end{array}$$

where R is the reflection of the sample. If the reflectance coefficient is considered to be constant, F is proportional to the absorption coefficient of the sample. The optical band gap was determined from the Tauc plot, i.e., from the cross point of linear plot sections in the coordinates (F·hν)^1/2^ = *f* (hν) corresponding to an allowed indirect transition.

#### SEM

The morphology of the samples was investigated by scanning electron microscopy (SEM) on the Zeiss Merlin scanning electron microscope with field emission cathode, electron optics column GEMINI-II and oil-free vacuum system. Energy-dispersive X-ray microanalysis (Oxford Instruments INCAx-act) was also carried out in order to detect the platinum co-catalyst.

#### BET

The specific surface area of the samples was determined using the Brunauer-Emmett-Teller (BET) method (Quadosorb SI) by measuring the amount of adsorbed nitrogen.

### Photocatalytic Experiments

#### Experimental Technique

**Figure 1:**
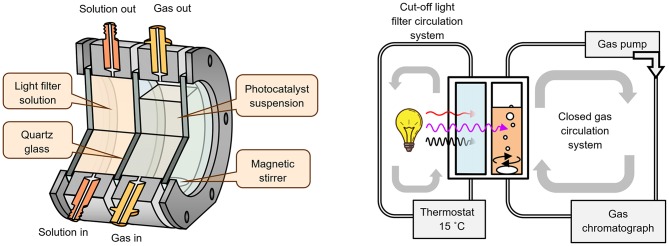
Photocatalytic reaction cell and scheme of the photocatalytic experimental set-up.

**Figure 2:**
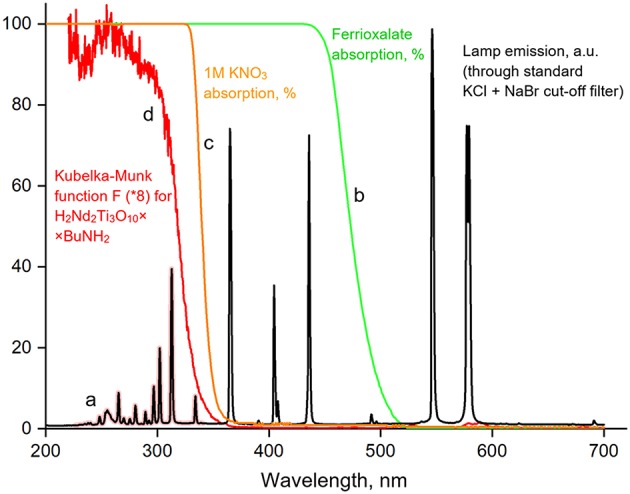
Emission spectrum for the DRT-125 lamp used for photocatalytic experiments (a), absorption of the light-sensitive potassium ferrioxalate solution used for light intensity measurements (b), absorption of the additional KNO_3_ cut-off filter (c), absorption of the H_2_Nd_2_Ti_3_O_10_×BuNH_2_ photocatalyst expressed by the Kubelka-Munk function (d).

The photocatalyst suspension (50 mL) was placed in an external-irradiation reaction cell (Figure 1), equipped with a magnetic stirrer, a liquid cut-off filter and connected to a closed gas circulation system (120 mL dead volume). A medium-pressure mercury lamp DRT-125 (125W) was used as a radiation source. Light reaches the reaction cell only after passing through a thermostated at 15°C light filter solution (KCl + NaBr, 6 g/L each, 2 cm optical path), which cuts off radiation with λ < 220 nm (Figure 2). During the photocatalytic reaction, hydrogen accumulates in the gas phase, which composition was analyzed by an on-line gas chromatograph at certain time intervals (Shimadzu GC-2014, Rt-Msieve 5A Column, TCD, Ar carrier). At the beginning of each experiment, the system was deaerated and argon gas was introduced at atmospheric pressure.

To prepare the suspension for the photocatalytic experiment, 30 mg of the photocatalyst sample were added to 60 mL of aqueous alcohol solution of desired concentration (the standard concentration was 1 mol.%). The suspension was shaken and left for 1 h to establish equilibrium between the photocatalyst and the solution. Straight before the experiment, each suspension was sonicated for 10 min (Elmasonic S10H ultrasound bath) to disaggregate the photocatalyst particles.

Platinum co-catalyst nanoparticles were loaded on the photocatalyst by the common method of photocatalytic platinization *in situ*. During the first experimental series, 1 ml of 2.56 mmol·L^−1^ H_2_PtCl_6_ aqueous solution was injected into the reaction suspension after the kinetic curve of hydrogen evolution for the bare sample was collected during 2 h. The concentration corresponds to 1 wt.% of Pt with respect to the catalyst, which was chosen as a standard amount. After H_2_PtCl_6_ was introduced into the reaction cell, the system was flushed with argon for 15 min under UV-irradiation, then the system was closed and the kinetic data were collected. Since hydrogen already evolves during these 15 min at quite a high rate, its amount at the starting point of measurement is non-zero. In the second experimental series, platinization was carried out at the beginning of the experiment without the 2 h pre-radiation period. In some experiments, the platinum content was varied in the range of 0.1–5 wt.% by the corresponding adjustment of the H_2_PtCl_6_ concentration.

#### Determination of the Apparent Quantum Efficiency

The apparent quantum efficiency of hydrogen generation ϕ was calculated by the formula

(6) $$\begin{array}{l} \varphi =\frac{\omega }{\omega_{0}} \end{array}$$

where ω is the observed hydrogen evolution rate measured in mol·h^−1^ and ω_0_ is the theoretical maximum hydrogen evolution rate, if we assume that all incident photons with energy greater than *E*_*g*_ are absorbed with the generation of electron-hole pairs, which subsequently lead to hydrogen reduction and alcohol oxidation with a 100% yield without recombination and other side-reactions. According to the half-reaction

(7) $$\begin{array}{l} 2{\text{H}}^{+}+2{\text{e}}^{-}={\text{H}}_{2} \end{array}$$

two electrons and thus two photons are needed to produce one hydrogen molecule. Therefore, ω_0_ equals half of the incident photon flux with λ < 340 nm (*I*_1_), which can be absorbed by the catalyst according to its bandgap. The required photon flux *I*_1_ was determined by a differential ferrioxalate actinometry method.

At the first step, we determined the photon flux with λ < 550 nm (*I*_2_) which corresponds to the absorption edge of ferrioxalate. For this aim we prepared the light-sensitive solution of potassium ferrioxalate (5 g) in 1 L of 0.1 M H_2_SO_4_, introduced 50 ml into the reaction cell and performed irradiation at the same conditions, as during photocatalytic experiments, but for shorter periods of time (10 and 20 s). The amount of generated Fe^2+^ ions was determined by the standard photometric method with the addition of 1,10-phenanthroline and acetate buffer. The calculated photon flux *I*_2_ was 32 mE·h^−1^.

At the second step, we replaced the usual light filter solution (NaBr + KCl) with a 1 M solution of KNO_3_, which completely cuts off light with λ < 340 nm (Figure 2). Then we performed the actinometric experiment again and thus determined the photon flux with 340 < λ < 550 nm (*I*_3_) which was found to be 17 mE·h^−1^. Finally, we calculated the required photon flux *I*_1_ by the difference between *I*_2_ and *I*_3_: 15 mE·h^−1^. This corresponds to a theoretical hydrogen evolution rate ω_0_ of 7.5 mmol·h^−1^.

Two remarks should be made about apparent quantum efficiency ϕ, determined by this approach. On the one hand, it is the lower estimate of the true quantum efficiency, because we assume 100% light absorption by the catalyst and do not take into account any scattering. On the other hand, the ϕ value is not limited by 100% because of the possible current doubling effect (Schneider and Bahnemann, 2013).

## Results and Discussion

### Characterization of Samples

**Figure 3:**
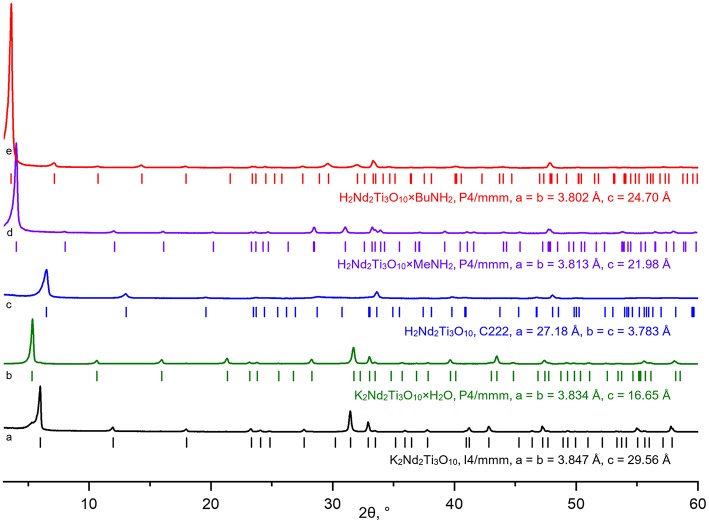
X-ray diffraction patterns and unit cell parameters of synthesized layered titanates K_2_Nd_2_Ti_3_O_10_ (a), K_2_Nd_2_Ti_3_O_10_×H_2_O (b), H_2_Nd_2_Ti_3_O_10_ (c) and amine-intercalated hybrid compounds H_2_Nd_2_Ti_3_O_10_×MeNH_2_ (d), H_2_Nd_2_Ti_3_O_10_×BuNH_2_ (e).

**Figure 4:**
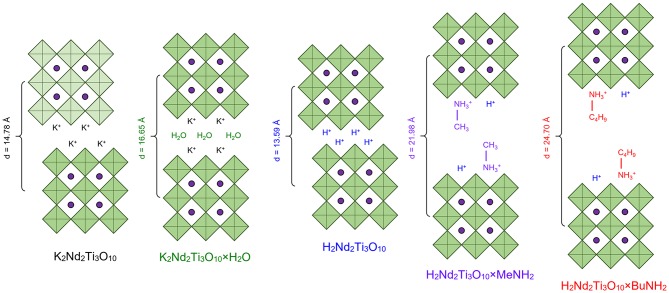
Schematic representation of the interlayer distance change at different synthetic steps.

According to powder XRD analysis (Figure 3), the layered oxide K_2_Nd_2_Ti_3_O_10_ was obtained as an almost single-phase product with a minor impurity of the hydrated form K_2_Nd_2_Ti_3_O_10_×H_2_O which is a result of its contact with atmospheric moisture. After prolonged contact with 75% humid air the sample completely transformed into the hydrated phase with enlarged interlayer distance *d* (Figure 4). The anhydrous form was indexed in the I4/mmm space group with *a* = 3.847 Å, *c* = 29.56 Å, which is close to the literature data (ICDD #01-087-0479). The hydrated form was indexed in P4/mmm with *a* = 3.834 Å, *c* = 16.65 Å.

**Figure 5:**
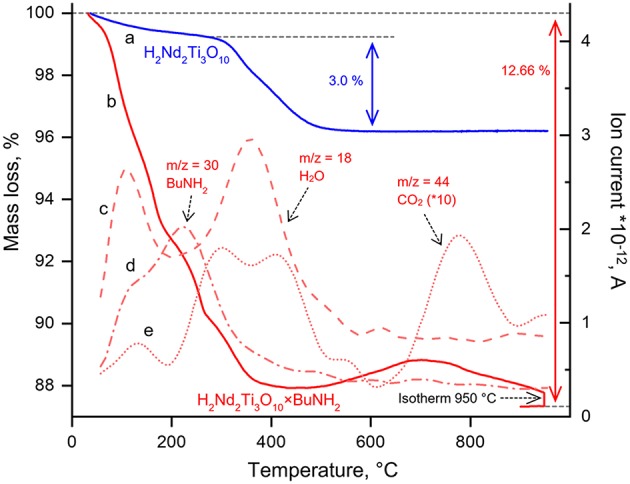
Thermogravimetric curves for H_2_Nd_2_Ti_3_O_10_ (a), H_2_Nd_2_Ti_3_O_10_×BuNH_2_ (b) and ion currents recorded during simultaneous thermal analysis coupled with mass spectrometry at m/z = 18 (c), m/z = 30 (d), m/z = 44 (e).

The ion exchange reaction in diluted hydrochloric acid led to a single-phase protonated compound H_2_Nd_2_Ti_3_O_10_ which was indexed in a C222 space group with *a* = 27.18 Å, *b* = *c* = 3.783 Å. The interlayer distance *d*, which in this case equals *a*/2, desreased in comparison to the initial alkaline form due to the exchange of potassium cations for more compact protons. Thermogravimetric analysis (Figure 5) revealed that the degree of protonation is 100% meaning that all potassium ions were successively substituted with protons. Also, the sample contained about 0.2 intercalated water molecules per formula unit, which are irreversible liberated at T > 150°C. Thus, its true composition can be expressed as H_2_Nd_2_Ti_3_O_10_·0.2H_2_O.

The treatment of the H_2_Nd_2_Ti_3_O_10_ sample with methylamine solution led to a significant increase in the interlayer distance, indicating the intercalation of methylamine molecules into the interlayer space, and further exchange reaction with *n*-butylamine led to even greater expansion of the interlayer gallery (Figure 4). The final product H_2_Nd_2_Ti_3_O_10_×BuNH_2_ was obtained as a single phase with tetragonal symmetry, which is perfectly indexed in the simple P4/mmm space group with parameters *a* = 3.802 Å, *c* = 24.70 Å.

Thermogravimetric analysis was carried out in order to determine the content of *n*-butylamine in the sample. The TG curve (Figure 5) demonstrates a significant mass loss from room temperature to 400°C followed by a mass increase up to 700°C and finally the loss of mass at higher temperatures. In order to explain such complex behavior, STA-MS was carried out. Mass spectrometry showed that at the first step (T < 200°C) butylamine and water are the main components which are liberated into the gas phase. Apparently, at this step we observe only the deintercalation of both compounds. At the second step (200°C < T < 500°C) not only water but also carbon dioxide is liberated, whereas the *n*-butylamine content in the gas phase gradually decreases. This may be associated with the beginning of the *n*-butylamine burning accompanied by the subsequent decomposition of the protonated H_2_Nd_2_Ti_3_O_10_ compound. At temperature about 500°C the mass of the sample begins to rise, that may be explained by the oxidation of either the remained carbon-containing species or the titanium cations, which might be reduced at lower temperatures by the reaction with organics. However, subsequent liberation of CO_2_ during the mass loss after 700°C clearly indicates, that some carbon species still remain in the sample after 500°C, when all butylamine is apparently gone. After an isotherm segment at 950°C for 20 min the mass of the sample stabilizes and an overall mass loss of 12.66% is reached. If we reasonably assume, that the final composition of the sample is described by the formula Nd_2_Ti_3_O_9_, then this mass loss gives us the upper estimate of the *n*-butylamine content in the sample, which is 0.9 per formula unit. While calculating this value, we also take into account the amount of H_2_O formed during H_2_Nd_2_Ti_3_O_10_ decomposition. However, the sample may also contain some unknown amount of intercalated water, which contributes to the mass loss at the first step. Thus, another independent method is needed to determine the composition of the sample more precisely. The use of CHN analysis data allowed to calculate the content of the organic component in the sample, which, together with thermogravimetric data, gave us following formula of the sample: H_2_Nd_2_Ti_3_O_10_·0.8BuNH_2_·0.4H_2_O. Also, CHN analysis showed that the molar ratio of C/N in this sample is close to 4, which indicates complete substitution of methylamine by *n*-butylamine.

**Figure 6:**
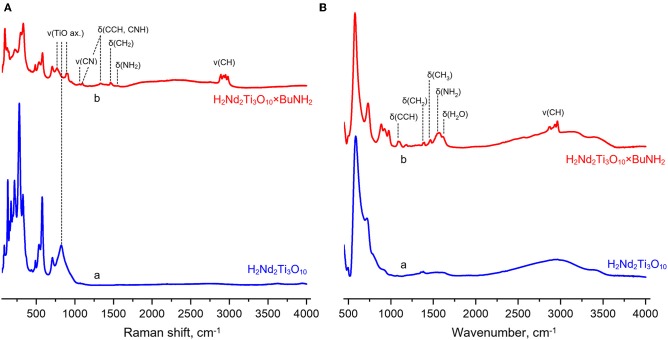
Raman **(A)** and infrared **(B)** spectra of the protonated titanate H_2_Nd_2_Ti_3_O_10_ (a) and its butylamine-intercalated form H_2_Nd_2_Ti_3_O_10_×BuNH_2_ (b).

Raman spectra of the protonated form and its *n*-butylamine derivative are presented in Figure 6. The hybrids formation is indicated by appearance of characteristic bands relating to latitudinal vibrations of C–C–H/C–N–H (1,100, 1,330 cm^−1^), methylene (1,460 cm^−1^) and amino fragments (1,575 cm^−1^) as well as stretching of C–N (1,055 cm^−1^) and C–H fragments (2,860–3,000 cm^−1^). Intercalation of *n*-butylamine is accompanied by redistribution of some bands' intensities (265–280 and 320 cm^−1^) and suppression of vibrations at 160–250 cm^−1^. The band relating to the symmetric stretching mode (ν_1_) of axial Ti–O bonds (810 cm^−1^ for the protonated forms) splits into two bands (765, 895 cm^−1^) during *n*-butylamine intercalation that points at the existence of two types of octahedra with unequal axial Ti–O distances.

IR spectra of the samples Figure 6 also indicate successful *n*-butylamine intercalation, the presence of water in the interlayer space (1,625 cm^−1^) and stretching of its O–H fragments (wide band at 2,800–3,500 cm^−1^). Thereby, IR spectroscopy indicates joint intercalation of *n*-butylamine and water. The absence of characteristic bands at 3,500–3,300 cm^−1^ relating to unprotonated amino groups –NH_2_ shows that *n*-butylamine is presented in the interlayer space in the form of *n*-butylammonium ions. This fact is well consistent with data from the earlier report (Tahara et al., 2007) where the presence of interlayer *n*-butylamine in the cationic form was confirmed using NMR spectroscopy.

**Figure 7:**
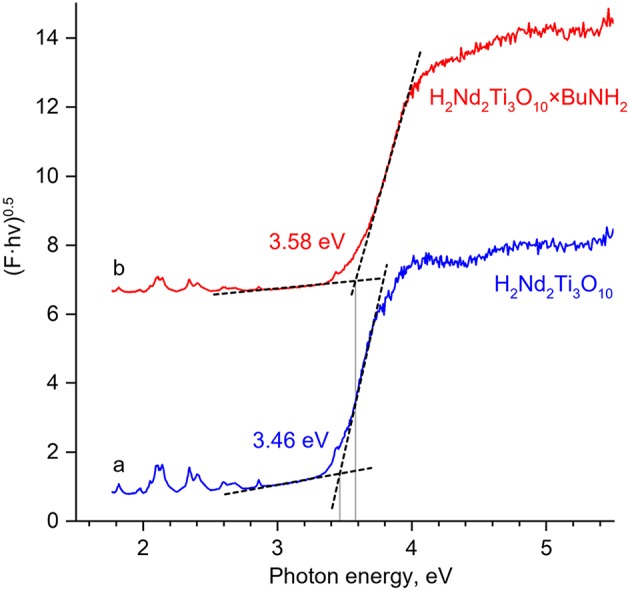
The Tauc plots for H_2_Nd_2_Ti_3_O_10_ (a) and H_2_Nd_2_Ti_3_O_10_×BuNH_2_ (b) based on diffuse reflectance measurements. The Kubelka-Munk function F is plot against the photon energy hν in the coordinates (F·hν)^0.5^ – hν. The bandgap energy is determined from the cross point of linear sections.

The bandgap energy of the butylamine-intercalated titanate (3.58 eV) slightly exceeds that of the initial H_2_Nd_2_Ti_3_O_10_ (3.46 eV) as can be seen from the Tauc plot of the Kubelka-Munk function (Figure 7). This is quite expected, because the bandgap usually increases with the increase of the interlayer distance in the absence of additional factors (Rodionov et al., 2017d). It is important, that due to the close values of band gap energy, both catalysts absorb the same peaks in the lamp emission spectrum (Figure 2), and thus there is no factor of different amount of available light, which could otherwise contribute to the difference in observed photocatalytic activity.

**Figure 8:**
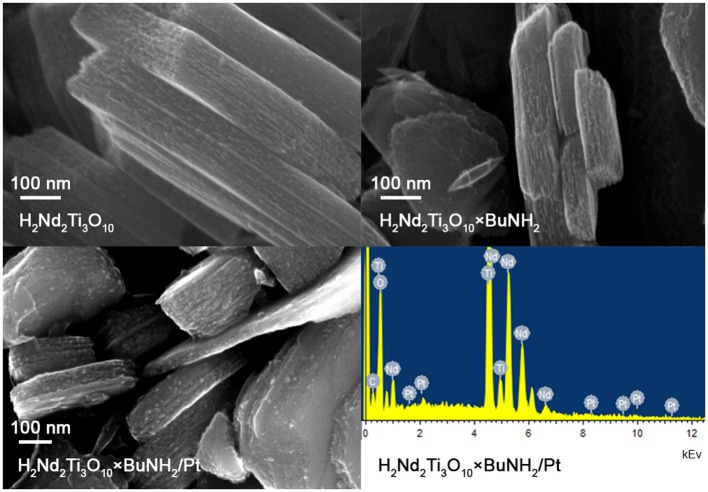
SEM images of initial H_2_Nd_2_Ti_3_O_10_ and H_2_Nd_2_Ti_3_O_10_×BuNH_2_ samples, SEM image and EDX spectrum for H_2_Nd_2_Ti_3_O_10_×BuNH_2_/Pt sample after the photocatalytic experiment in 1% methanol solution.

SEM investigation showed that the particle morphology of H_2_Nd_2_Ti_3_O_10_×BuNH_2_ is almost the same as for the initial H_2_Nd_2_Ti_3_O_10_ (Figure 8). We observe plate-like particles with an irregular size in the range of 100–1,000 nm that is typical for layered oxides prepared by the solid-state method. The morphology also does not considerably change after the photocatalytic experiment with platinization. The platinum nanoparticles can be observed at the SEM image as light dots with an average diameter of 4–6 nm. The EDX analysis also confirms the presence of 1 ± 0.4 wt.% platinum in this sample, which is consistent with the expected value.

The BET surface area of the H_2_Nd_2_Ti_3_O_10_ sample was found to be 21 m^2^/g. Unfortunately, we were unable to measure the surface area for amine-intercalated samples due to the fact that they are unstable under vacuum conditions. However, the preservation of the particle morphology allows to assume that at least the outside surface area of the samples does not change much as a result of intercalation.

### Photocatalytic Activity

**Figure 9:**
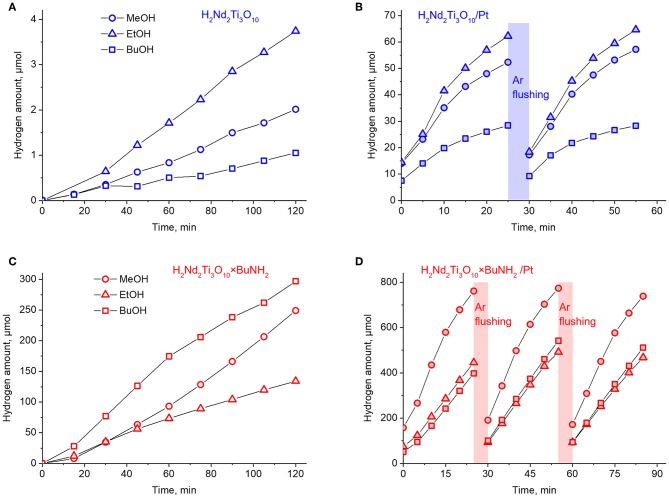
Kinetic curves of photocatalytic hydrogen evolution over H_2_Nd_2_Ti_3_O_10_ **(A)**, H_2_Nd_2_Ti_3_O_10_/Pt **(B)**, H_2_Nd_2_Ti_3_O_10_×BuNH_2_ **(C)**, and H_2_Nd_2_Ti_3_O_10_×BuNH_2_/Pt **(D)**.

The results of photocatalytic experiments are presented in Figure 9. In each case the hydrogen evolution rate ω was calculated from the slope of the kinetic curve. In case when the curve is not linear, the initial rate was calculated from the first points by linear approximation. These data, as well as the calculated values of apparent quantum efficiency ϕ, are summarized in Table 1. The standard error of hydrogen evolution rate determined in this way is estimated as 7%.

**Table 1:** Rate of photocatalytic hydrogen evolution (ω) and its apparent quantum efficiency at 220–340 nm (ϕ) from 1 mol.% aqueous methanol, ethanol and *n*-butanol solutions over H_2_Nd_2_Ti_3_O_10_, H_2_Nd_2_Ti_3_O_10_/Pt, H_2_Nd_2_Ti_3_O_10_×BuNH_2_ and H_2_Nd_2_Ti_3_O_10_×BuNH_2_/Pt.

**Photocatalyst**	**Methanol**		**Ethanol**		***n*****-Butanol**	
	**ω, mmol/h**	**ϕ, %**	**ω, mmol/h**	**ϕ, %**	**ω, mmol/h**	**ϕ, %**
H_2_Nd_2_Ti_3_O_10_	0.0011	0.015	0.0020	0.027	0.00057	0.0076
H_2_Nd_2_Ti_3_O_10_/Pt	0.12	1.6	0.15	2.0	0.064	0.86
H_2_Nd_2_Ti_3_O_10_×BuNH_2_	0.12	1.7	0.077	1.0	0.20	2.6
H_2_Nd_2_Ti_3_O_10_×BuNH_2_/Pt	1.7	23	1.0	14	1.1	15

The protonated titanate H_2_Nd_2_Ti_3_O_10_ demonstrates a very low photocatalytic activity with a quantum efficiency of hydrogen generation in the range of 0.01–0.03% depending on the alcohol nature. After 1 wt.% platinum was loaded on it as a co-catalyst, the hydrogen evolution rate significantly increased and the quantum efficiency reached 2% in the case of ethanol. This is quite expected because platinum nanoparticles serve both for the improved charge separation and as active hydrogen evolution sites. Note that both the platinized and non-platinized H_2_Nd_2_Ti_3_O_10_ showed the maximum hydrogen evolution rate for ethanol rather than methanol. Probably, this is due to the fact that ethanol is a slightly stronger reducing agent.

For the platinized sample the hydrogen evolution rate decreases with time, but restores its original value after flushing the gas system with argon. Thus, we can conclude that the deceleration is associated with the accumulation of hydrogen in the gas phase which may give rise to some backward reactions involving hydrogen and alcohol oxidation products (i.e., aldehydes) as reactants.

After *n*-butylamine was introduced into the interlayer space, the photocatalytic activity of the bare titanate increased to almost the same level as for the platinized non-intercalated sample in the case of methanol. It is known, that the interlayer space plays an important role in the reaction of water splitting by alkaline forms of triple-layered titanates A_2_Ln_2_Ti_3_O_10_ (A = alkaline metal, Ln = La, Nd). Remarkable activity is observed only for K- and Rb-containing compounds, which are capable of spontaneous water intercalation, whereas Li- and Na-containing compounds do not undergo water intercalation and show almost no activity. A similar relationship is also noticed for triple-layered Dion-Jacobson phases. Thus, the increased photocatalytic activity may be explained by the expansion of the interlayer space by *n*-butylamine, which allows the alcohol substrate molecules to intercalate and undergo oxidation between the perovskite layers. Surprisingly, in this case the maximum quantum efficiency of 2.6% was observed for *n*-butanol, which showed the worst performance for initial H_2_Nd_2_Ti_3_O_10_. The reason is not quite clear yet, but it may be due to the similarity of *n*-butanol and *n*-butylamine molecules, which facilitates the incorporation of alcohol molecules in the interlayer space during the reaction.

Finally, after platinization *in situ* the photocatalytic activity of H_2_Nd_2_Ti_3_O_10_×BuNH_2_ increased by one order of magnitude. This time the best quantum efficiency (23%) was achieved in the case of methanol, whereas ethanol and *n*-butanol gave 14–15%. The effect of reaction rate decay with time is also noted here, but it is not as significant as in the previous case. Thus, the effects of *n*-butylamine intercalation and platinization together gave rise to a much higher efficiency, than any of them separately. The reason is probably that these two modification methods affect different parts of the photocatalytic system and thus different reaction steps. While *n*-butylamine is likely to promote the alcohol oxidation in the interlayer space, the platinum co-catalyst particles facilitate the hydrogen reduction on the surface. Since during photocatalysis the oxidation and reduction reactions should proceed at the same rate, if only one of them is promoted, then the other may become rate-limiting. But if both are promoted, the rate of the whole process increases much more efficiently.

During the photocatalytic experiment with H_2_Nd_2_Ti_3_O_10_×BuNH_2_/Pt in 1 mol.% methanol solution hydrogen was formed with a total amount of ca. 2.5 mmol. The mass of photocatalyst in each experiment was 25 mg. Thus, even if we assume the content of *n*-butylamine in the sample to be equal to its upper limit according to TGA data (0.9 per formula unit), the total amount of *n*-butylamine in the reaction system is only 0.034 mmol. That means a more than 70-fold excess of hydrogen was generated without significant loss of reaction rate. Therefore, we can conclude that the reaction proceeds catalytically and that hydrogen is formed from the reaction solution rather than from the intercalated amine.

**Figure 10:**
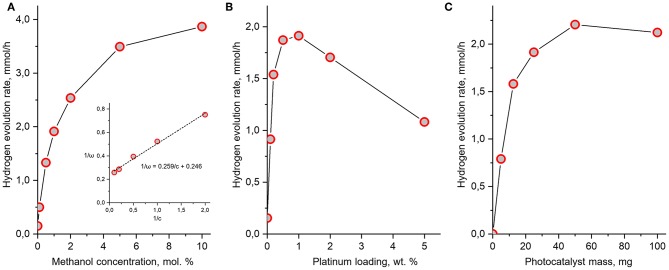
Dependence of hydrogen evolution rate over H_2_Nd_2_Ti_3_O_10_×BuNH_2_/Pt on methanol concentration **(A)**, platinum loading **(B)**, and photocatalyst mass **(C)**. Unless varied, the methanol concentration is 1 mol.%, the platinum content is 1 wt.% and the mass of photocatalyst is 25 mg in every experiment.

Since the maximum efficiency was observed for H_2_Nd_2_Ti_3_O_10_×BuNH_2_/Pt in methanol solution, we decided to investigate this photocatalytic system more extensive. Particularly, we studied the effect of different operating conditions, such as methanol concentration, amount of platinum loaded and the catalyst amount on the hydrogen evolution rate. We stated 1 mol.% methanol concentration, 1 wt.% platinum loading and 25 mg photocatalyst mass as standard conditions and varied one of these parameters in each experiment series. Unlike in previous cases, this time we added H_2_PtCl_6_ straight at the beginning of the experiment to obtain the platinized photocatalyst immediately. The results are presented at Figure 10.

Usually, the dependence of photocatalytic reaction rate ω on the substrate concentration *c* is described by the Langmuir-Hinshelwood equation

(8) $$\begin{array}{l} \omega =\frac{kKc}{Kc+1} \end{array}$$

where *k* is the apparent rate constant, *K* is the adsorption constant of the substrate by the catalyst. If this is the case, a linear relation between ω^−1^ and c^−1^ should be present. As can be seen from Figure 10, such a relation between the hydrogen evolution rate and methanol concentration is indeed observed. Like usual, the reaction order with respect to methanol gradually decreases with the increase of its concentration. However, in our case the Langmuir-Hinshelwood equation is obeyed only at methanol concentrations higher than 0.5 mol.%. At lower concentrations there are deviations caused by the fact that the hydrogen evolution rate from water without methanol is not zero, but has a significant value of 0.15 mmol/h (ϕ = 2%). Since methanol is not the only reactant which can act as a source of hydrogen, but there is also water, the kinetics of the overall reaction cannot be fully described by the simple Equation (8).

At standard conditions (1 mol.% methanol) the measured hydrogen evolution rate was 1.9 mmol/h that is somewhat greater, than in the first experimental series (1.7 mmol/h). This is due to the fact that the platinization was performed immediately at the start of the experiment thus preventing any possible undesired processes, which may occur with the bare catalyst during the first 2 h of experiment, such as partial degradation *n*-butylamine or sedimentation.

The maximum hydrogen evolution rate (3.9 mmol/h) was observed at the maximum methanol concentration (10 mol.%) and corresponds to an apparent quantum efficiency as high as 52%.

The dependence of photocatalytic activity on the amount of loaded platinum co-catalyst represents a curve with a maximum around 1 wt.%. Such type of dependence has been reported earlier for many photocatalytic systems, including layered oxides (Kudo et al., 1988; Cui et al., 2006; Rodionov et al., 2014), but the position of the maximum always depends on the nature of the photocatalyst and co-catalyst. Thus, our standard platinum content of 1 wt.% was found to be optimal accidentally. The slow decay of hydrogen evolution rate at higher platinum content is explained by the increased light shielding of the catalyst by the platinum particles.

Finally, the observed dependence of the hydrogen evolution rate on the catalyst amount is also consistent with known examples (Minero and Vione, 2006). At low catalyst concentrations a linear dependence is observed due to the light absorption being proportional to the suspension turbidity. When the catalyst concentration is so high that almost no light transmits through the suspension, the dependence reaches saturation and the change of catalyst amount does not considerably affect the reaction rate. If the catalyst concentration is set even higher, a slow decay of reaction rate is observed due to increased light scattering. In the conditions of our experiment, the maximum efficiency is observed around 50 mg of catalyst and it is only 15% higher than at the standard amount of 25 mg. The optimal catalyst concentration is therefore 1 g/L, which is consistent with literature data for other systems.

## Conclusions

We have discovered, that the intercalation of *n*-butylamine is an efficient method to improve the photocatalytic activity of the protonated triple-layered titanate H_2_Nd_2_Ti_3_O_10_ in the reaction of hydrogen production from aqueous solutions of methanol, ethanol and *n*-butanol. The obtained hybrid photocatalyst H_2_Nd_2_Ti_3_O_10_×BuNH_2_/Pt demonstrated an apparent quantum efficiency of 52% in the wavelength range of 220–340 nm. The hydrogen evolution rate was stable with time and the amount of generated hydrogen exceeded the amount of intercalated *n*-butylamine more than 70 times, indicating that hydrogen is produced from the alcohol solution rather than from the intercalated amine. The dependences of photocatalytic activity on operating conditions, such as alcohol concentration, platinum co-catalyst content and catalyst loading are well-described by the usual theoretical approaches for heterogeneous photocatalysis. We propose that the intercalation of amines is an effective strategy to improve photocatalytic properties of protonated layered oxides that can be applied to other related compounds as well. At the present moment we investigate carefully the composition, structure and properties of the samples which are obtained after photocatalytic experiments, including the platinized samples. It is important to explore what changes occur in the interlayer space during the reaction. Also, further studies involving different amines and different layered oxides are in progress.

## Data Availability Statement

The datasets generated for this study are available on request to the corresponding author.

## Author Contributions

IR, OS, and IZ contributed the conception and design of the study. Experimental work was carried out by EM (photocatalytic experiments, quantum efficiency), SK and AP (synthesis, characterization) under supervision of IR and OS. IR wrote the manuscript and prepared images with contributions of OS, EM, and IZ in certain sections. All authors participated in the analysis and discussion of obtained results.

### Conflict of Interest

The authors declare that the research was conducted in the absence of any commercial or financial relationships that could be construed as a potential conflict of interest.
